# Low concentrations of transforming growth factor-beta-1 induce tubulogenesis in cultured mammary epithelial cells

**DOI:** 10.1186/1471-213X-7-7

**Published:** 2007-02-08

**Authors:** Roberto Montesano, Fabio Carrozzino, Priscilla Soulié

**Affiliations:** 1 Department of Cell Physiology and Metabolism, University of Geneva Medical School, CH-1211 Geneva 4, Switzerland

## Abstract

**Background:**

Formation of branching tubes is a fundamental step in the development of glandular organs. To identify extracellular cues that orchestrate epithelial tubulogenesis, we employed an in vitro assay in which EpH4-J3B1A mammary epithelial cells form spheroidal cysts when grown in collagen gels under serum-free conditions, but form branching tubules in the presence of fetal calf serum (FCS).

**Results:**

Initial experiments showed that the tubulogenesis-inducing activity of FCS was markedly increased by heating (70°C) or transient acidification to pH3. We therefore hypothesized that the tubulogenic agent was transforming growth factor-beta (TGF-beta), a cytokine that is present in serum in latent form and can be activated by heat or acid treatment. We found indeed that the tubulogenic activity of acidified FCS is abrogated by addition of either SB-431542, a selective inhibitor of the TGF-beta type I receptor, or a neutralizing antibody to TGF-beta-1. On the other hand, addition of low concentrations (20–100 pg/ml) of exogenous TGF-beta-1 recapitulated the effect of acidified FCS in inducing morphogenesis of hollow tubes. In contrast, higher concentrations of TGF-beta-1 induced the formation of thin cellular cords devoid of a detectable lumen. To gain insight into the mechanisms underlying TGF-beta-1-induced tube formation, we assessed the potential role of matrix metalloproteinases (MMPs). By western blot and gelatin zymography, we observed a dose-dependent increase in MMP-9 upon TGF-beta-1 treatment. Tube formation was suppressed by a synthetic broad-spectrum metalloproteinase inhibitor, by recombinant tissue inhibitor of metalloproteinases-2 (TIMP-2) and by a selective inhibitor of MMP-9, indicating that this morphogenetic process requires the activity of MMP-9.

**Conclusion:**

Altogether, our results provide evidence that, at low concentrations, TGF-beta-1 promotes MMP-dependent branching tubulogenesis by mammary epithelial cells in vitro, and suggest that it plays a similar role during mammary gland development in vivo.

## Background

Formation of branched tubes from an initially unbranched epithelial bud is a fundamental morphogenetic process in the development of many organs, including pancreas, mammary gland, lung, and kidney [1,2]. Although the cellular and molecular mechanisms of tubulogenesis are still incompletely understood, a number of polypeptide growth factors have been shown to stimulate the formation and branching of epithelial tubes [3]. The most thoroughly characterized of these tubulogenic cytokines are hepatocyte growth factor/scatter factor (HGF/SF) [4-6], glial cell-derived neurotropic factor [7,8], and several members of the fibroblast growth factor family [9,10].

Elucidation of the mechanisms responsible for epithelial tubulogenesis is made difficult by the multiplicity and complexity of cell interactions occurring in vivo. To overcome this drawback, several groups including our own have designed three-dimensional cell culture systems that accurately recapitulate key events of tubulogenesis, thereby facilitating its molecular analysis [11]. The recent development of an experimental model in which EpH4-J3B1A mammary epithelial cells form spheroidal cysts when grown in collagen gels in chemically defined medium [12] has provided an additional tractable assay for deciphering the constellation of signals that govern branching tubulogenesis.

Transforming growth factor-β (TGF-β) is the prototypic member of a superfamily of structurally related cytokines involved in the regulation of a broad spectrum of biological processes, including cell proliferation, differentiation, apoptosis, production of extracellular matrix, and tissue repair. Three TGF-β isoforms (referred to as TGF-β1, TGF-β2 and TGF-β3) have been described in mammals. TGF-βs are secreted as inactive complexes, in which the C-terminal mature homodimer is non-covalently bound to a dimer of its N-terminal precursor polypeptide, also known as latency associated peptide (LAP). The LAP, in turn, is disulfide-bonded to an unrelated protein, which is referred to as latent TGF-β binding protein (LTBP). TGF-β activation, i.e. the release of TGF-β from LAP, may be mediated by different mechanisms and represents a critical step in the regulation of TGF-β bioactivity [13]. TGF-βs achieve their pleiotropic activities through the activation of heteromeric complexes of transmembrane serine/threonine kinase receptors designated as TGF-β type I (TβRI) and type II (TβRII) receptors. Ligand binding to TβRII induces the recruitment and transphosphorylation of TβRI. Activated TβRI phosphorylates receptor-associated Smads (Smad2 and Smad3), which then bind Smad4 and translocate to the nucleus, where they regulate transcription of target genes. In addition to Smads, other signaling pathways, including mitogen-activated protein kinases (MAPK), can also be activated by TGF-βs [14-18].

Herein, we report that low concentrations (20–100 pg/ml) of TGF-β1 rapidly induce tube formation in cultured mammary epithelial cells, and that this biological response requires MMP activity.

## Results

### A heat- and acid-resistant factor in bovine serum stimulates branching tubulogenesis of EpH4-J3B1A mammary epithelial cells

This study was prompted by the finding that addition of FCS to serum-free collagen gel cultures of J3B1A cells [12], a clonal derivative of the murine EpH4 mammary epithelial cell line [19-21], stimulates the formation of branching tubes. When grown in collagen gels in chemically-defined medium supplemented with all-*trans*-retinoic acid, J3B1A cells formed spheroidal cysts enclosing a patent lumen [12]. Addition of 10% FCS to preformed cysts induced the radial outgrowth of tube-like structures from the cyst wall. FCS-induced tube formation was more pronounced in gels that had been released and allowed to float in the medium (R. Montesano, unpublished data), possibly owing to the greater compliance of unrestrained collagen matrices [22,23]. Floating gels were therefore used for the remainder of this study.

**Figure 1:**
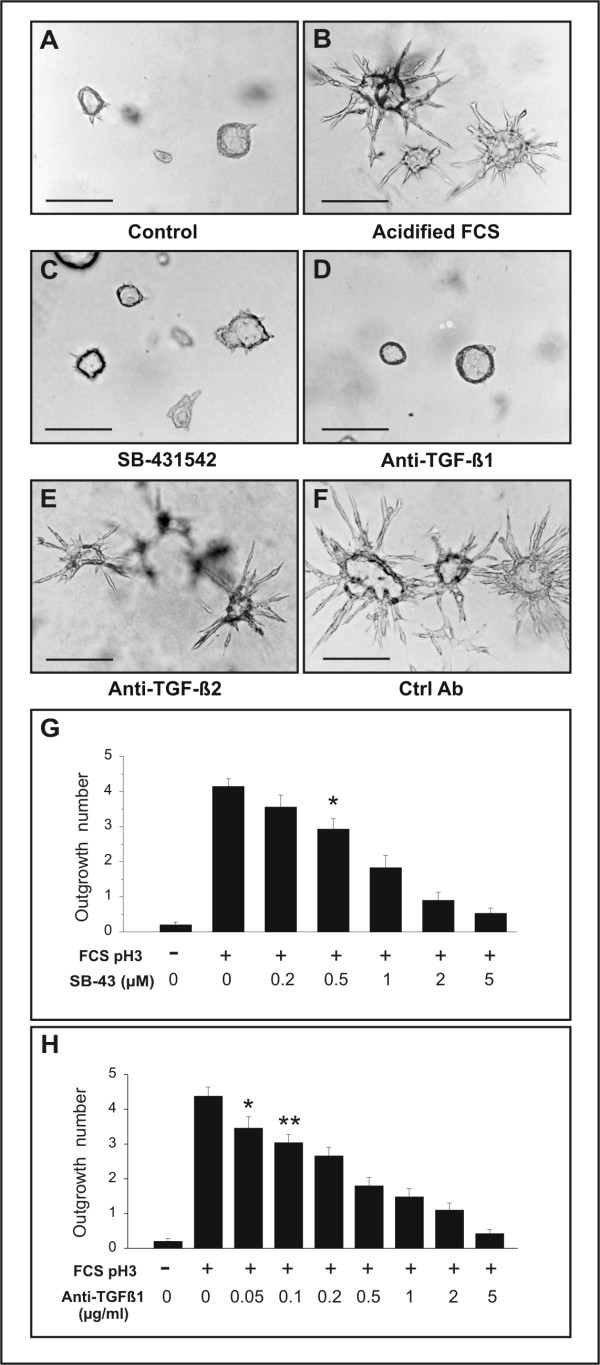
TGF-β is the acid-activated serum factor that induces branching tubulogenesis. J3B1A cells were grown in collagen gels in defined medium for 6 days to allow the formation of cystic structures. The cultures were then left untreated or were incubated with 1% acidified FCS (pH 3) for an additional 48 hours. (A) Under control conditions, J3B1A cells form spheroidal cysts enclosing a wide lumen. (B) Addition of acidified FCS induces the radial outgrowth of tube-like structures from the cyst wall. (C) Co-addition of 5 μM SB-431542, a selective inhibitor of the TGF-β type I receptor, abolishes the tubule-inducing activity of acidified FCS. (D) Pre-incubation of acid-treated FCS with a neutralizing antibody specific for TGF-β1 abrogates the tubulogenic effect of acidified FCS, whereas pre-incubation with a neutralizing antibody to TGF-β2 (E) or with a control antibody that does not react with TGF-βs (F) have no inhibitory effect. Antibodies in D-F were added at a final concentration of 5 μg/ml. Bars, 200 μm. (G) Branching tubulogenesis is suppressed by SB-431542 in a dose-dependent manner. The cultures were treated with the inhibitor two hours before addition of 1% acidified FCS. Data were expressed as mean number of outgrowths per colony ± s.e.m. from three separate experiments and statistical significance was determined using the Student's unpaired *t*-test. * p < 0.0025 versus values of acidified FCS alone. (H) Dose-response analysis of the effect of increasing concentrations of anti-TGF-β1 antibody. Acidified FCS (1%) was pre-incubated for 60 minutes with the indicated concentrations of the antibody before being added to the cultures. * p < 0.025 versus values of acidified FCS alone. ** p < 0.0005 versus values of acidified FCS alone.

The ability of FCS to stimulate formation of branching tubes in collagen gel cultures of J3B1A cells suggested the existence of a putative tubulogenic factor (or factors) in serum. Preliminary experiments aimed at characterizing the physico-chemical properties of the serum factor(s) showed that the tubulogenesis-inducing activity of FCS was not only resistant to heat, but was in fact enhanced by heating (70°C, 10 min; data not shown). The tubulogenic activity was also markedly increased by transient acidification of FCS to pH 3. Thus, addition of as little as 1% acidified FCS to J3B1A cells grown in collagen gels in defined medium elicited a vigorous tubulogenic response within only 24–48 hours (Fig. 1A,B).

### The tubulogenic component in FCS is TGF-β1

Based on the foregoing findings, we considered the possibility that the tubulogenic factor was TGF-β, a cytokine that is present in serum in latent form and can be activated by heat or acid treatment [24-26]. To test this hypothesis, we first assessed whether pre-treatment with SB-431542, a selective inhibitor of the TGF-β type I receptor [27,28], would prevent the tubulogenic effect of acidified FCS. We found that branching tubulogenesis was suppressed by SB-431542 in a dose-dependent manner (Fig. 1C,G). To further validate the hypothesis that the tubulogenic agent in FCS is TGF-β, and to determine the potential TGF-β isoform responsible for induction of branching tubulogenesis, acid-treated FCS was pre-incubated with isoform-specific neutralizing antibody to either TGF-β1 or TGF-β2 before being added to J3B1A cells in collagen gels. Anti-TGF-β1 antibodies abrogated the tubulogenic effect of acidified FCS in a dose-dependent manner, a significant inhibition (p < 0.025) being observed with concentrations of antibody as low as 50 ng/ml (Fig. 1D,H). In contrast, neither a function-blocking anti-TGF-β2 antibody (Fig. 1E), nor a control antibody that does not react with TGF-βs (Fig. 1F) inhibited the tubulogenic activity of acidified FCS. These results clearly identified TGF-β1 as the tubulogenic agent present in FCS.

### Low concentrations of exogenous TGF-β1 recapitulate the tubulogenic effect of acidified FCS

**Figure 2:**
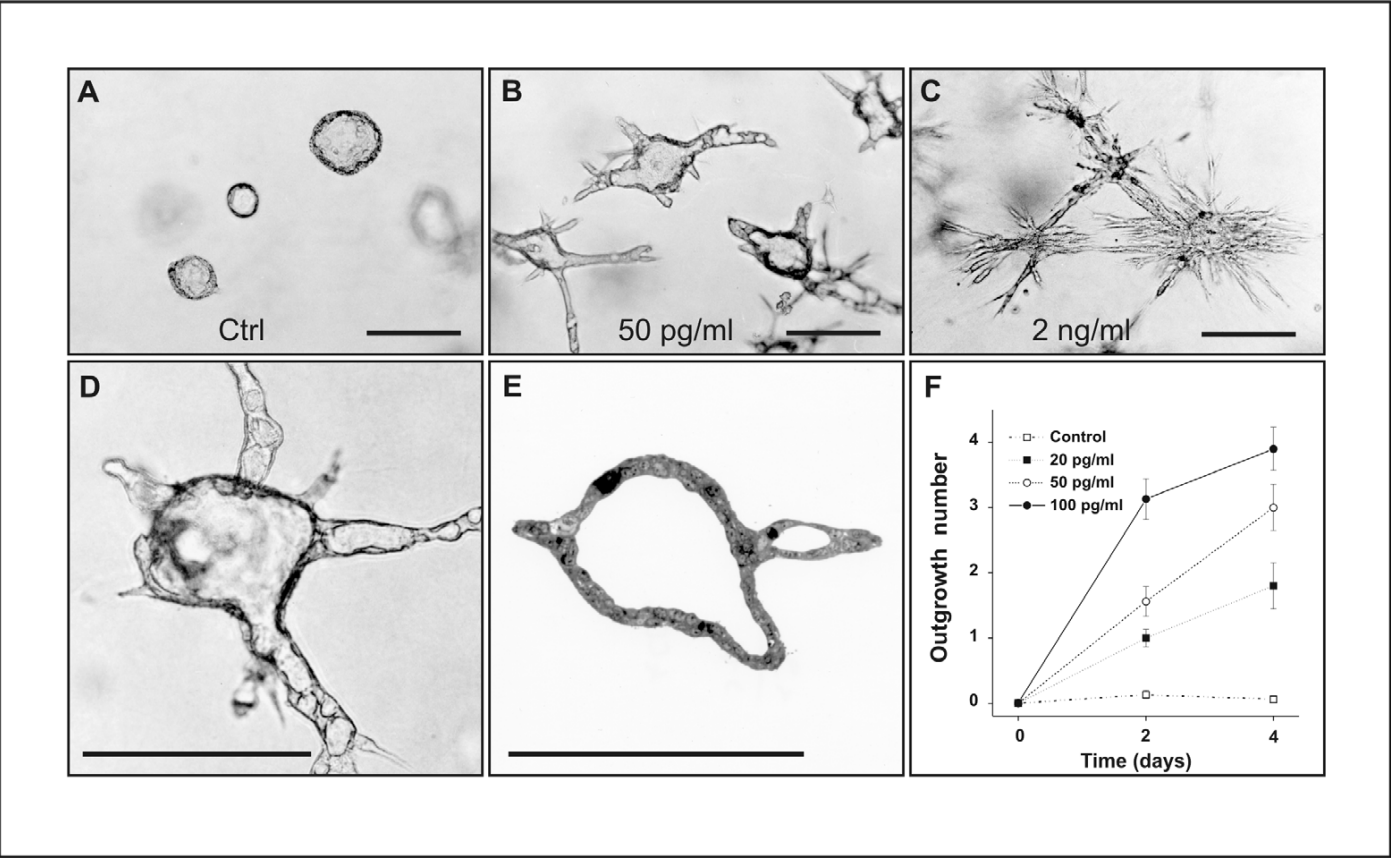
Low concentrations of exogenous TGF-β1 induce morphogenesis of branching tubules. (A) J3B1A cells grown in a collagen gel in defined medium for a total of 10 days. (B) Parallel culture in which J3B1A cells were grown in a collagen gel for 6 days to allow cyst formation and were subsequently treated with 50 pg/ml TGF-β1 for an additional 4 days. TGF-β1 has induced the outgrowth of tube-like structures from the wall of existing cysts. (C) Treatment with 2 ng/ml TGF-β1 has resulted in the formation of numerous thin cell cords extending out into the surrounding collagen matrix. Notably, at this relatively high concentration, TGF-β1 also disrupts the organization of preformed cysts, resulting in lumen obliteration. (D) Higher magnification view of a multicellular structure formed in a culture treated with 20 pg/ml TGF-β1 for 4 days. The outgrowths enclose a patent lumen, which at least in some tubes is continuous with the cavity of the cyst. (E) Semi-thin section of a collagen gel culture of J3B1A cells treated with 50 pg/ml TGF-β1 for 4 days. Bars (A-E), 200 μm. (F) Quantitative analysis of TGF-β1-induced tubulogenesis. J3B1A cells were grown in a collagen gel for 6 days to allow cyst formation and were subsequently treated with different concentrations of TGF-β1. Tube formation was evaluated as described in Materials and Methods after 4 days of treatment. Data were expressed as mean number of outgrowths per colony ± s.e.m. from three separate experiments. p < 0.0005 for values of 20 pg/ml TGF-β1 at 2 days compared with control at 2 days, as well as for values of 100 pg/ml TGF-β1 at 2 days compared with 50 pg/ml TGF-β1 at 2 days; p < 0.0025 for values of 50 pg/ml TGF-β1 at 4 days compared with 2 days; p < 0.01 for values of 50 pg/ml TGF-β1 at 4 days compared with 20 pg/ml TGF-β1 at 4 days; p < 0.025 for values of 50 pg/ml TGF-β1 at 2 days compared with 20 pg/ml TGF-β1 at 2 days, as well as for values of 20 pg/ml TGF-β1 at 4 days compared with 2 days.

We next asked whether exogenous TGF-β1 could be able to recapitulate the tubulogenic activity of acid-treated FCS. As TGF-β1 is known to elicit different cellular responses in the same cell type depending on its concentration, we examined the effects of a wide range of concentrations of TGF-β1. Addition of low concentration (20 to 100 pg/ml) of recombinant TGF-β1 stimulated the rapid formation of branching tubular outgrowths from the wall of the original cysts (Fig. 2B). At higher magnification, the outgrowths were seen to enclose a patent lumen, which was often continuous with the cyst cavity (Fig. 2D). Examination of semi-thin sections confirmed the tubular nature of TGF-β1-induced centrifugal outgrowths (Fig. 2E). The tubulogenic effect of TGF-β1 was already evident after 24 hours and increased progressively during the next 2–3 days of treatment. At slightly higher concentrations (200–500 pg/ml), TGF-β1 induced the extension of pointed outgrowths devoid of a visible lumen (not shown). Finally, incubation with TGF-β1 at concentrations greater than 500 pg/ml resulted in the conversion of existing cysts into disorganized, lumen-less cellular aggregates, from which numerous thin cellular cords extended radially into the surrounding matrix (Fig. 2C). Similar effects were observed following addition of recombinant TGF-β2 or TGF-β3 (data not shown). These results indicated that when added to collagen gel cultures of J3B1A cells in the concentration range of 20–100 pg/ml, exogenous TGF-βs induce the morphogenesis of hollow tubular structures that are reminiscent of mammary gland ducts. Based on the foregoing findings, subsequent experiments were designed to characterize in more detail the tubulogenic activity of low concentrations of TGF-β1. A quantitative analysis demonstrated that TGF-β1 elicits the formation of tubular outgrowths in a dose- and time-dependent manner, a significant (p < 0.0005) effect being observed after 48 hours of treatment with TGF-β1 concentrations as low as 20 pg/ml (Fig. 2F).

**Figure 3:**
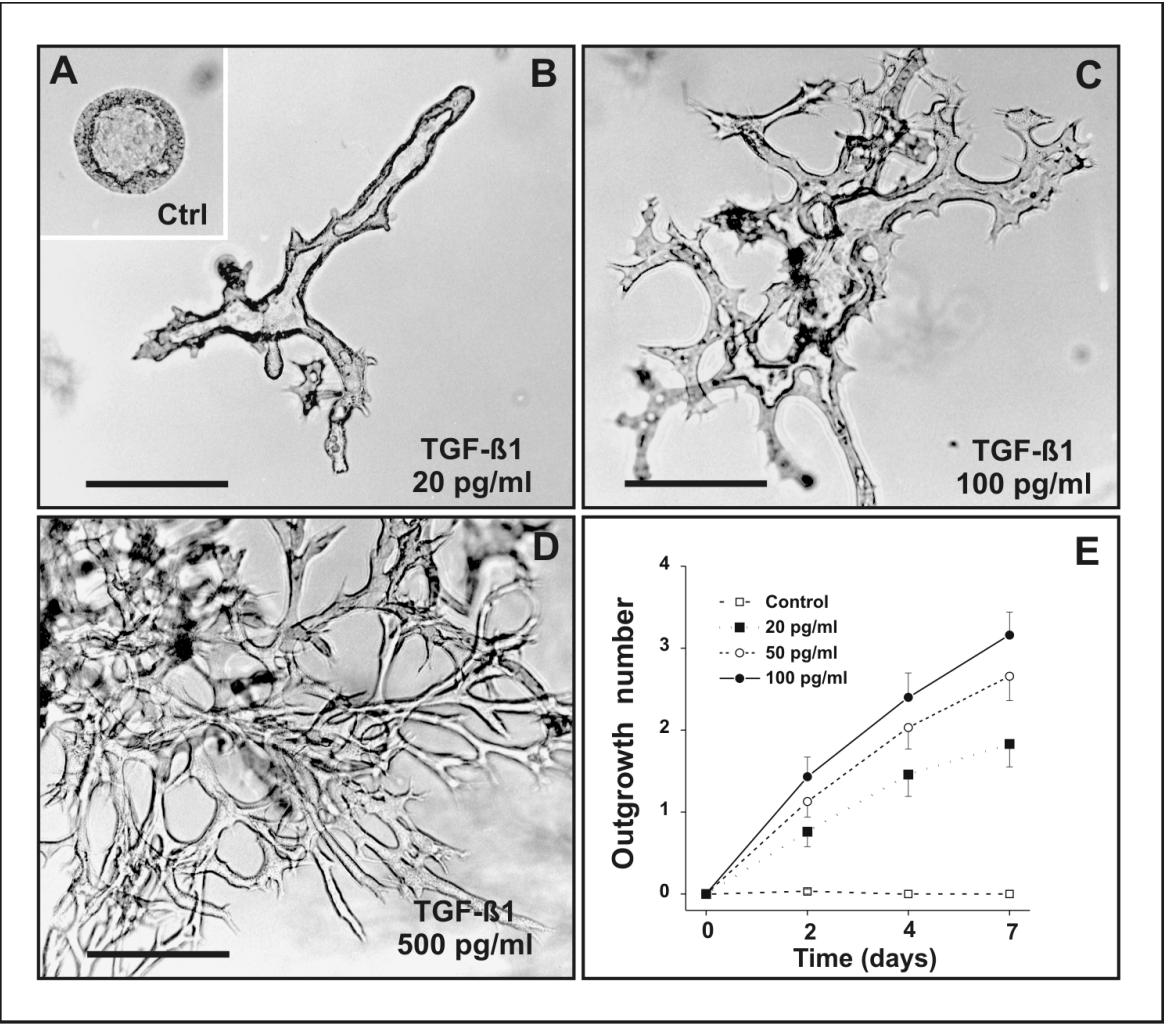
TGF-β1-induced tubulogenesis in fibrin gels. (A) J3B1A cells grown in a fibrin gel in defined medium for 11 days have formed spherical cysts. (B, C) J3B1A cells grown in a fibrin gel in defined medium for 4 days and subsequently treated with 20 pg/ml (B) or 100 pg/ml (C) TGF-β1 for an additional 7 days have formed branched tubules. (D) Cells grown as in (A-C) but incubated with 500 pg/ml TGF-β1 have generated a complex network of solid anastomosing cords. Bar, 200 um. (E) Quantitative analysis of TGF-β1-induced tubulogenesis in fibrin gels. J3B1A cells were grown in a fibrin gel for 6 days to allow cyst formation and were subsequently treated with different concentrations of TGF-β1. Tube formation was evaluated as described in Materials and Methods after 2, 4 and 7 days of treatment. Data were expressed as mean number of outgrowths per colony ± s.e.m. from three independent experiments. p < 0.0005 for values of 20 pg/ml TGF-β1 at 2 days compared with control at 2 days, as well as for values of 50 pg/ml and 100 pg/ml TGF-β1 at 7 days compared with 2 days; p < 0.005 for values of 20 pg/ml TGF-β1 at 7 days compared with 2 days; p < 0.01 for values of 50 pg/ml TGF-β1 at 4 days compared with 2 days; p < 0.025 for values of 100 pg/ml TGF-β1 at 4 days compared with 2 days; p < 0.05 for values of 20 pg/ml TGF-β1 at 4 days compared with 2 days, as well as for values of 50 pg/ml TGF-β1 at 2 days compared with 20 pg/ml TGF-β1 at 2 days.

To determine whether TGF-β1-induced tubulogenesis was dependent on cell interaction with collagen fibrils or could also occur in other types of three-dimensional matrices, we next examined the TGF-β1 response of J3B1A cells suspended in fibrin gels. When grown in fibrin gels in defined medium, J3B1A cells formed spherical cysts (Fig. 3A). In striking contrast, in the presence of 20–100 pg/ml TGF-β1, the cells formed branched tubules (Fig. 3B,C). Addition of TGF-β1 at concentrations greater than 200 pg/ml induced the development of complex, highly arborized networks of branching and anastomosing cell cords (Fig. 3D), which were however devoid of a visible lumen, similarly to what we had observed in collagen gels (see above).

A quantitative analysis demonstrated that TGF-β1 elicits tube formation in fibrin gels in a dose- and time-dependent manner, a highly significant (p < 0.0005) effect being observed after 48 hours of treatment with TGF-β1 concentrations as low as 20 pg/ml (Fig. 3E). Finally, we assessed the effect of TGF-β1 on J3B1A cells grown in Matrigel, a laminin-rich extracellular matrix [29]. Contrary to what we observed in collagen or fibrin gels, both control and TGF-β1-treated cells formed spheroidal colonies in Matrigel. The lack of tube formation in this experimental setting is unlikely due to an inhibitory effect of a Matrigel component, because J3B1A cells exhibited a robust tubulogenic response to TGF-β1 (50 pg/ml) when grown in a 1:1 mixture of Matrigel and type I collagen (data not shown). We speculate that cells suspended in pure Matrigel, which is a highly compliant non-fibrillar substratum [30], are unable to generate the traction forces required for centrifugal outgrowth into the surrounding matrix.

### Inhibitors of p38 MAPKs abrogate TGF-β1-induced tubulogenesis

**Figure 4:**
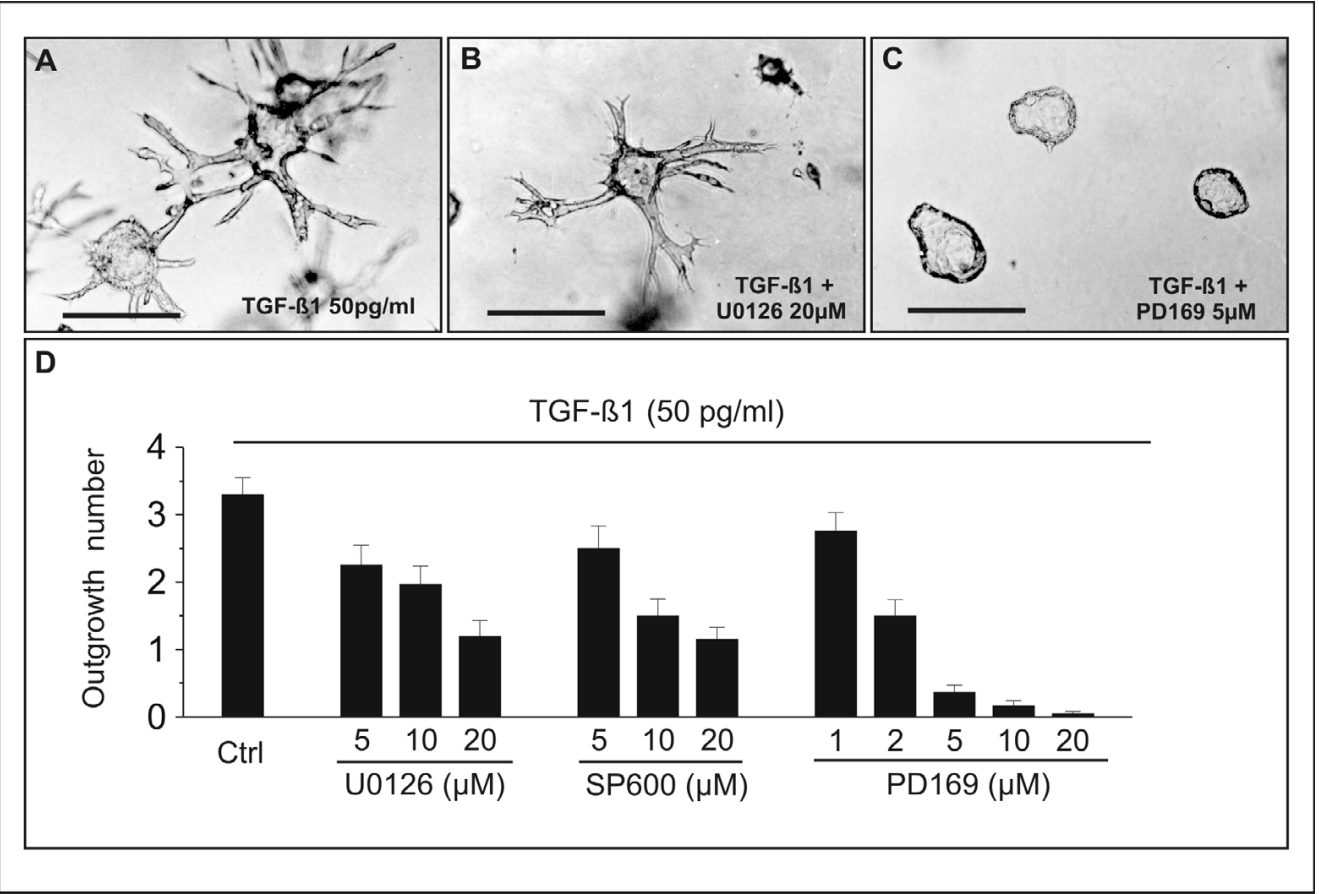
Suppression of TGF-β1-induced tubulogenesis by a p38 inhibitor. (A-C) J3B1A cells were grown in collagen gels for 6 days and subsequently treated with 50 pg/ml TGF-β1 alone (A), co-treated with TGF-β1 and U0126, an inhibitor of the ERK activators MEK1 and MEK2 (B), or co-treated with TGF-β1 and PD169316, a p38 inhibitor (C), for 4 days. The inhibitors were added two hours before treatment with TGF-β1. Whereas relatively high concentrations (20 μM) of U0126 only attenuate TGF-β1-induced tubulogenesis, PD169316 has a profound inhibitory effect at a concentration as low as 5 μM. Bars, 200 μm. (D) Quantitative analysis of inhibition of tubulogenesis. Data were expressed as mean number of outgrowths per colony ± s.e.m. from at least three separate experiments, and statistical significance was determined using the Student's unpaired *t*-test. p < 0.01 and p < 0.0005 for values of 5 μM and 10 μM U0126, respectively, compared with cultures incubated with TGF-β1 alone (Ctrl). p < 0.05 and p < 0.0005 for values of 5 μM and 10 μM SP 600125, respectively, compared with cultures incubated with TGF-β1 alone (Ctrl). p < 0.0005 for values of 2 μM PD169316 compared with cultures incubated with TGF-β1 alone (Ctrl).

In addition to the Smad-mediated signaling pathway, mounting evidence indicates that TGF-β also activates several mitogen-activated protein kinases (MAPKs), including extracellular signal-regulated kinases (ERKs), c-Jun N-terminal kinases (JNKs) and p38 kinases [31-34]. To begin assessing the contribution of MAPKs to TGF-β-induced branching tubulogenesis, we used several small-molecule inhibitors that selectively block the ERK, JNK, or p38 pathways. Pretreatment of collagen gel cultures with U0126, a selective inhibitor of the ERK activators MEK1 and MEK2 [35], attenuated TGF-β1-induced branching tubulogenesis in a dose-dependent manner, a 63% inhibition being observed at 20 μM (Fig. 4B,D). Likewise, addition of SP600125, a JNK inhibitor [36], resulted in 65% inhibition of tubulogenesis at 20 μM (Fig. 4D). Remarkably, the p38 inhibitor PD169316 [37] was comparatively much more effective in suppressing TGF-β1-induced tubule formation, with an 88% inhibition being observed with a concentration as low as 5 μM and a virtually total inhibition at 20 μM (Fig. 4C,D). These findings suggest that MAPK (and particularly p38) signaling is required for TGF-β1-induced tubulogenesis. Validation of this hypothesis will await additional studies involving knockdown of MAPKs by siRNA technology.

### TGF-β1-induced branching tubulogenesis requires MMP activity

**Figure 5:**
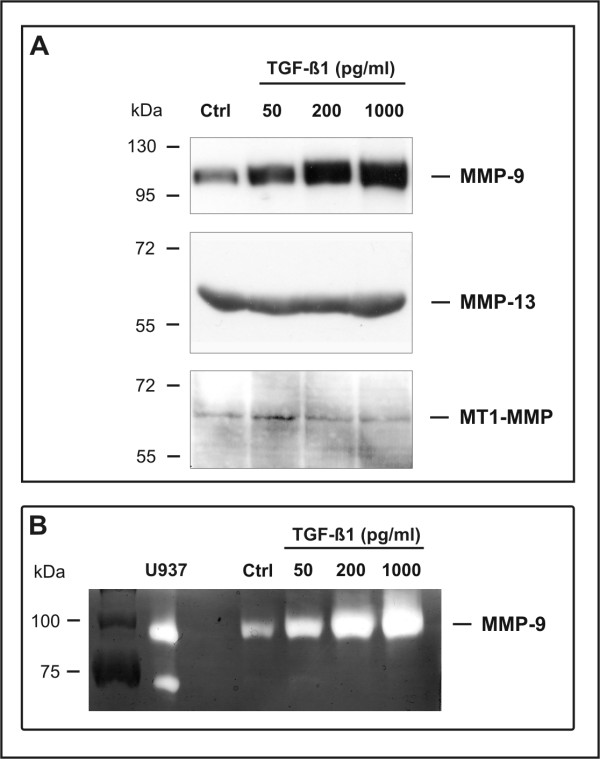
TGF-β1 induces the production of MMP-9 by J3B1A cells. (A) Western blot analysis of conditioned media from J3B1A cells incubated with 50, 200 and 1000 pg/ml TGF-β1 for 72 hours. MMP-9 is induced by TGF-β1 in a dose-dependent manner. MMP-13 and MT1-MMP (MMP-14) are constitutively expressed by J3B1A cells and are not ostensibly modulated by TGF-β1. Conditioned medium from PMA-treated human U937 cells was used as a positive control for MMP-9, and conditioned medium from SVEC4-10 cells as a control for MMP-13 and MMP-14. Uniform loading of lanes was verified by silver staining. The blots shown are representative of at least two independent experiments. (B) Gelatin zymography of conditioned media from J3B1A cells incubated with 50, 200 and 1000 pg/ml TGF-β1 for 72 hours. TGF-β1 induces the secretion of MMP-9 in a dose-dependent manner, a clear increase in MMP-9 activity being already evident at 50 pg/ml TGF-β1. Conditioned medium from PMA-treated U937 cells, which are known to produce MMP-2 and MMP-9, was used as a positive control.

Formation of epithelial tubes in collagen gels has previously been shown to require the activity of matrix metalloproteinases (MMPs) [38-41]. To assess the potential role of MMPs in TGF-β1-induced tubulogenesis, we examined the effect of TGF-β1 on the production of MMPs implicated in extracellular matrix (ECM) degradation, including membrane-type-1-MMP (MT1-MMP or MMP-14), 72 kDa gelatinase (MMP-2), 92 kDa gelatinase (MMP-9), and collagenase-3 (MMP-13) [42]. By Northern blot, MT1-MMP (MMP-14) mRNA was constitutively expressed by J3B1A cells but not ostensibly modulated by TGF-β1, whereas MMP-2 mRNA was not detected. In contrast, the levels of MMP-9 mRNA were increased by TGF-β1 in a dose-dependent manner (data not shown). Western blot analysis confirmed both the lack of modulation of MT1-MMP (MMP-14) and the dose-dependent induction of MMP-9 (Fig. 5A). Western blots also showed that MMP-13 is constitutively expressed by J3B1A cells, and that this expression is not ostensibly modulated by TGF-β1 (Fig. 5A). By zymographic analysis [43], conditioned media from untreated J3B1A cells produced a band of gelatin lysis corresponding to the reported molecular weight (102–105 kDa) of the latent form of mouse MMP-9. Treatment with TGF-β1 resulted in a marked dose-dependent increase in this activity. In contrast, consistent with the lack of detection of MMP-2 mRNA, no proteolytic activity corresponding to the molecular weight of MMP-2 was detected in control or TGF-β1-treated cells (Fig. 5B).

**Figure 6:**
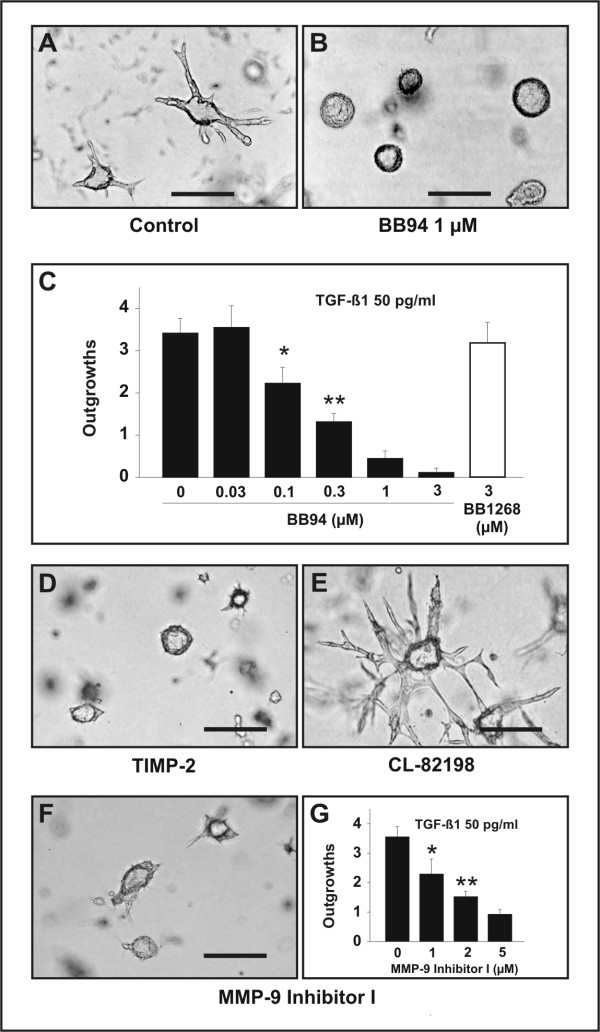
Effect of MMP inhibitors on TGF-β1-induced tubulogenesis. (A-C) The broad spectrum metalloproteinase inhibitor BB94 abrogates TGF-β1-induced branching tubulogenesis. J3B1A cells were grown in a collagen gel for 6 days to allow cyst formation and were subsequently treated with 50 pg/ml TGF-β1 for an additional 4 days in the absence (A) or in the presence (B) of BB94 (1 μM; the inhibitor was added two hours before treatment with TGF-β1). Bars, 200 μm. (C) Quantitative analysis of the effect of BB94 on TGF-β1-induced tubulogenesis. J3B1A cells grown in collagen gels for 6 days were treated with TGF-β1 alone (50 pg/ml), co-treated with TGF-β1 and BB94 (30 nM to 3 μM), or co-treated with TGF-β1 and the inactive isomer BB1268 (3 μM) for 4 days. Formation of tubular outgrowths is suppressed in a dose-dependent manner by BB94, but is not significantly decreased by the inactive isomer BB1268. * p < 0.0125 and ** p < 0.0005 compared with cultures incubated with TGF-β1 alone. (D-F) TGF-β1-mediated branching tubulogenesis is suppressed by pre-treatment with 2 μg/ml recombinant TIMP-2 or 5 μM MMP-9 Inhibitor I, but not by pre-treatment with 100 μM CL-82198, a selective inhibitor of MMP-13. Bars, 200 μm. (G) Quantitative analysis of the effect of MMP-9 inhibitor I. J3B1A cells grown in collagen gels for 6 days were treated with TGF-β1 alone (50 pg/ml) or co-treated with TGF-β1 and MMP-9 Inhibitor I (1–5 μM) for 4 days (MMP-9 Inhibitor I was added two hours before treatment with TGF-β1). Formation of tubular outgrowths is abrogated by MMP-9 inhibitor I in a dose-dependent manner. * p < 0.0025 and ** p < 0.0005 compared with cultures incubated with TGF-β1 alone.

To determine whether MMP activity is required for TGF-β1-induced tubulogenesis, we first assessed the effect of TGF-β1 (50 pg/ml) in the presence of the hydroxamate-based metalloproteinase inhibitor BB94 [44]. Formation of branching tubules was abrogated in a dose-dependent manner by BB94, but not by the related inactive isomer BB1268 (Fig. 6A–C). Since hydroxamate-based inhibitors also inactivate members of the adamalysin family of metalloproteinases [45,46], we next used recombinant tissue inhibitor of metalloproteinases-2 (TIMP-2), which more selectively suppresses the activity of MMPs [47]. TIMP-2 (2 μg/ml) inhibited TGF-β1-induced tubulogenesis (Fig. 6D) by 84.6% (p < 0.0005; n = 30 colonies from three independent experiments), thereby confirming the involvement of the MMP family of metalloproteinases. Finally, we examined the potential effect of CL-82198 and MMP-9 Inhibitor I, two synthetic compounds that selectively target MMP-13 and MMP-9, respectively [48,49]. Addition of CL-82198 at concentrations ranging from 10 to 100 μM had no obvious inhibitory activity on TGF-β1-induced tubulogenesis (Fig. 6E), whereas MMP-9 Inhibitor I suppressed this biological response in a dose-dependent manner (Fig. 6F,G). These findings support a role for MMP-9 in TGF-β1-induced branching tubulogenesis.

## Discussion

To identify the molecular cues that orchestrate epithelial tubulogenesis, we took advantage of an in vitro system in which J3B1A mammary epithelial cells grown in collagen gels in chemically defined medium form spherical cysts. Addition of acidified FCS to the defined medium induced the formation of branching tubes. Using a pharmacological inhibitor of TGF-β receptor signaling and a neutralizing antibody to TGF-β1, we identified the active component in acidified FCS as TGF-β1. Importantly, the effect of acidified FCS was accurately recapitulated by exogenous TGF-β1 in the concentration range of 20–100 pg/ml. These findings demonstrate that, at low concentrations, TGF-β1 can activate a morphogenetic program culminating in formation of epithelial tubes.

TGF-β1 is a multifunctional cytokine that elicits different and sometimes opposite cellular responses in the same cell type depending on its concentration [50-54]. In accordance with this notion, the effects of TGF-β1 in our system were clearly concentration-dependent. Thus, in the range of 20–100 pg/ml, TGF-β1 stimulated the rapid extension of hollow branched tubes from the wall of existing cysts. In contrast, at concentrations higher than 200 pg/ml, TGF-β1 induced the formation of thin cellular outgrowths, similar to those described in cultures of gallbladder [55] and thyroid [56] epithelial cells.

The results presented here complement those obtained previously using TAC-2 cells, a different murine mammary epithelial cell line [57]. In the latter study, we reported that low concentrations of TGF-β1 promote the elongation and branching of epithelial cords. However, the absence of a central lumen within TAC-2 cell cords supported the interpretation that TGF-β1 was sufficient to mediate only a subset of morphogenetic events involved in the formation of duct-like structures [57]. Using J3B1A mammary epithelial cells grown in chemically defined medium, we provide now evidence that, at concentrations of 20–100 pg/ml, TGF-β1 is able to induce the formation of hollow tubes that mimic the organization of mammary gland ducts.

Several groups have reported that TGF-β1, at relatively high concentrations, stimulates a process of epithelial-mesenchymal transition (EMT) characterized by the acquisition of spindle-like cell morphology, reorganization of cortical actin into stress fibers, downregulation and/or relocalization of junctional proteins (e.g., E-cadherin, β-catenin and ZO-1) and gain of mesenchymal markers [33,58,59]. This phenotypic conversion has been primarily described in NMuMG mammary epithelial cells, but is much less pronounced, or does not occur at all, in other cell lines. Thus, in MCF-10A mammary epithelial cells, TGF-β1 decreases the insoluble pool of E-cadherin without inducing conversion to a spindle phenotype [60]. Moreover, in a screening of 18 established epithelial cell lines, only two strains were reported to undergo EMT in response to TGF-β1 [61]. Of particular relevance to the present study, it has been shown [20] that TGF-β1 induces full EMT in Ras-transformed EpH4 cells, but not in wild-type EpH4 cells (i.e., the parental cell line from which we isolated the J3B1A clone). In our own studies, the effects of TGF-β1 on J3B1A cells grown on a planar substrate (e.g., in conventional plastic wells or atop of a collagen gel) were concentration-dependent. Following incubation with relatively high concentrations (200 pg/ml to 1 ng/ml) of TGFβ1 for 3 days, J3B1A cells acquired an irregular shape and underwent a moderate degree of scattering. In contrast, when treated with tubulogenic concentrations (e.g., 50 pg/ml) of TGF-β1, J3B1A cells formed multicellular colonies that were slightly less compact than in control cultures but nonetheless retained close cell-cell contacts and typical epithelial morphology (R. M., unpublished data). Likewise, we found that 1 ng/ml TGF-β1 induces the expression of mesenchymal markers (e.g., fibronectin and α-SM-actin) and slightly decreases the expression of epithelial markers (e.g., E-cadherin and tight-junction-associated proteins) in J3B1A cells. In contrast, 50 pg/ml TGF-β1 did not elicit ostensible changes in the epithelial markers examined, while inducing a marginal upregulation of α-SM-actin (F. C., unpublished data).

The concentration-dependent effects of TGF-β reported in this study can be interpreted as reflecting graded changes in epithelial plasticity. It is now well established that the epithelial phenotype is highly dynamic and finely modulated by microenvironmental cues. Epithelial plasticity encompasses a whole spectrum of changes, ranging from full EMT to more subtle alterations that are not associated with overt mesenchymal conversion [62,63]. Thus, during biological processes that involve coordinate cell repositioning, such as branching morphogenesis [64,65], epithelial cells transiently downregulate or relocalize adhesion proteins while remaining connected by intercellular junctions [11,41,66-68]. In light of these notions, we propose that low concentrations of TGF-β induce a moderate increase in epithelial plasticity, thereby facilitating the morphogenetic cell rearrangements required for tubulogenesis. In contrast, higher concentrations of TGF-β would trigger more pronounced alterations of the epithelial phenotype, resulting in disturbances of cell polarity and partial EMT. This hypothesis is supported by the finding that, in our system, low concentrations of TGF-β1 promote the development of well-organized tubular structures, while higher concentrations cause the formation of lumen-less cell cords.

The formation and elongation of tube-like epithelial structures in collagen gels involve a process of invasive growth [6,39], which is dependent on the activity of MMPs, a family of zinc-dependent endopeptidases that play a key role in ECM degradation [42]. As a first step toward elucidating the mechanisms responsible for TGF-β1-induced tubulogenesis, we assessed the effect of TGF-β1 on the production of MMPs involved in collagen turnover, including MT1-MMP (MMP-14), collagenase-3 (MMP-13), 72 kDa gelatinase (MMP-2) and 92 kDa gelatinase (MMP-9) [42]. MMP-13 and MMP-14 were constitutively expressed by J3B1A cells, but were not obviously modulated by TGF-β1. Interestingly, however, in accordance with previous studies in keratinocytes [69], corneal epithelial cells [70] and MCF-10 mammary epithelial cells [71], TGF-β1 markedly enhanced the expression of 92 kDa gelatinase (MMP-9) at the level of mRNA, protein, and enzymatic activity. While gelatinases are unable to cleave intact fibrillar collagen, they nonetheless play an important role in collagen degradation by acting sequentially after the initial cleavage of the triple helix by either MT1-MMP or interstitial collagenases [72]. Notably, MMP-9 is particularly well suited to effect pericellular proteolysis owing to its ability to bind to cell surface molecules [73,74]. To determine whether MMP activity is required for epithelial tube formation, we used the synthetic metalloproteinase inhibitor BB94 and the physiological MMP inhibitor TIMP-2, and found that both of them abrogate TGF-β1-induced tubulogenesis. Notably, a relatively selective inhibitor of MMP-9 (MMP-9 Inhibitor I) [49] significantly attenuated TGF-β1-induced tube formation, but was less efficient in this respect than the broad spectrum inhibitor BB94. These findings suggest that MMP-9 participates in TGF-β1-induced tubulogenesis in our system, possibly by acting in concert with other MMPs, and strengthen the notion that tube formation in collagen gels is dependent on MMP activity [38-41]. The molecular mechanisms underlying MMP requirement for TGF-β1-induced tube formation are not known. A straightforward possibility is that expression of MMPs at the advancing tip of the cellular outgrowths creates a path in the surrounding matrix, thereby facilitating directional tube elongation. However, MMPs might also participate in epithelial tubulogenesis by more complex mechanisms, e.g. by exposing cryptic sites in collagen molecules, by releasing proteolytic fragments of matrix proteins that stimulate cell motility, or by processing cell surface ECM receptors [42]. Overall, our study lends significant support to the notion [75-80] that the balance of MMP/MMP inhibitors has a key role in branching morphogenesis. It is likely, however, that this morphogenetic process involves additional mechanisms, including deposition of endogenous ECM components and altered expression of integrin or non-integrin matrix receptors. Further studies will be required to address these issues.

An important question raised by this study is whether the morphogenetic activity of TGF-β1 observed in our in vitro model is relevant to the process of branching tubulogenesis that occurs in vivo. At first sight, the finding that TGF-β1 induces tubule formation appears difficult to reconcile with previous in vitro and in vivo studies suggesting a negative regulatory role for TGF-βs in branching morphogenesis. It is noteworthy, however, that while most experimental evidence points to an inhibitory activity of TGF-βs [81-86], studies in embryonic lung have shown that low concentrations of TGF-β2 promote branching morphogenesis, whereas higher concentrations are inhibitory [87]. The prevalent notion that TGF-βs are negative modulators of branching morphogenesis is based on three main sets of experimental data. First, delivery of TGF-β1 to mouse mammary glands using slow release plastic implants has been shown to inhibit duct elongation [88]. It is possible however that relatively high concentrations of TGF-β1 were locally released by the implants, resulting in suppression of epithelial growth. In a second experimental approach, addition of exogenous TGF-β to lung bud organ cultures was reported to inhibit branching morphogenesis [82,85]. In those studies, however, high concentrations (1–100 ng/ml) of TGF-β were used. In a third approach, perturbation of TGF-β receptor signalling in vitro [83] or in vivo [86] was associated with increased epithelial branching, implying an inhibitory role for TGF-βs in branching morphogenesis. In those experimental settings, however, TGF-β-dependent signal transduction could have been attenuated rather than totally abrogated, thereby unmasking a potential morphogenic activity of low-level TGF-β signalling. Therefore, while caution needs to be exercised in extrapolating information gained from our in vitro system to the whole organism, the results reported here underscore the need for re-evaluating the in vivo effects of TGF-β on branching morphogenesis using a wide range of cytokine concentrations.

Parenchymal organs contain significant quantities of latent TGF-β stored in the ECM. As a consequence, TGF-β bioavailability is primarily regulated by the conversion of latent TGF-β to its active form. Interestingly, it has been shown that TGF-β activation in the mammary gland is controlled by ovarian hormones [89]. It is therefore tempting to speculate that spatially and temporally restricted activation of a small fraction of matrix-bound latent TGF-β contributes to the morphogenesis of mammary gland ducts.

In an attempt to reconcile the disparate and apparently paradoxical effects of TGF-β1 reported in the literature, we propose that TGF-β1 has multiple, context- and concentration-dependent effects on mammary gland epithelial cells. Low concentrations of the cytokine may increase epithelial plasticity and promote morphogenetic cell rearrangements that culminate in the development of branching ducts. At higher concentrations, the growth inhibitory activity of TGF-β1 is likely to override its ability to enhance epithelial plasticity, resulting in suppression of duct formation. Finally, sustained high-level expression of TGF-β1 in the setting of a genetically altered epithelium could lead to EMT, thereby fostering tumor progression.

## Conclusion

While the precise role of TGF-β1 in the regulation of branching morphogenesis in vivo remains to be assessed, our study clearly demonstrates that low concentrations of TGF-β1 can profoundly modify the spatial organization of epithelial cells, resulting in the formation of branching tubular structures. Owing to the rapidity and robustness of the morphogenetic response induced by TGF-β1, the experimental system we have developed affords a unique opportunity for dissecting the molecular and cellular mechanisms responsible for epithelial tubulogenesis. This system also provides a convenient bioassay with which to identify additional morphoregulatory molecules.

## Methods

### Reagents

Human platelet TGF-β1, TGF-β2 and TGF-β3 were purchased from R&D Systems Ltd. (Minneapolis, MN). Recombinant human epidermal growth factor (EGF) was from PeproTech (London, UK). ITS+ Premix was from BD Bioscences (Bedford, MA). All-*trans*-retinoic acid (RA) was purchased from Sigma Chemical Co. (St. Louis, MO), dissolved in DMSO, stored at -20°C and protected from light exposure. SB-431542 was obtained from Tocris Cookson Ltd. (Bristol, UK). SP600125 (JNK inhibitor-II), UO126, and PD169316 were purchased from either Calbiochem-Merck (Darmstadt, Germany) or Alexis Biochemicals (Carlsbad, CA). The synthetic broad spectrum MMP inhibitor BB94 and the related inactive isomer BB1268 were kindly provided by Dr. P. Brown (British Biotech Pharmaceuticals Ltd., Oxford, UK). MMP-9 Inhibitor I (Cat. No. 44278), the selective MMP-13 inhibitor CL-82198 (Cat. No. 233105) and recombinant tissue inhibitor of metalloproteinases-2 (TIMP-2, cat. No. PF021) were from Calbiochem-Merck. Chicken neutralizing antibody (IgY) to TGF-β1 (Cat. AB-101-NA), control chicken IgY that do not react with TGFβ1 (Cat. AB-101-C), and goat neutralizing antibody to TGF-β2 (Cat. AB-112-NA) were purchased from R&D Systems.

### Cells

J3B1A cells [12], a clonal derivative of the murine EpH4 mammary epithelial cell line [19-21], were grown in Dulbecco's modified Eagle's medium (DMEM, GIBCO Invitrogen Ltd, Bern, Switzerland) supplemented with 10% donor calf serum (DCS, GIBCO) and 2 mM L-glutamine.

### Assays of branching tubulogenesis

J3B1A cells were harvested with trypsin/EDTA from confluent cultures, centrifuged, and washed in serum-free DMEM/F12 medium (1:1). The cells were centrifuged once again and resuspended in a serum-free, chemically defined medium consisting of DMEM/F12, ITS+ Premix (6.25 μg/ml insulin, 6.25 μg/ml transferrin, 6.25 ng/ml selenious acid, 1.25 mg/ml bovine serum albumin and 5.35 μg/ml linoleic acid), 2 ng/ml EGF and 5 nM all-*trans*-retinoic acid (this medium will hereafter be referred to as "defined medium"). The cells were mixed with a type I collagen solution prepared as described [39] to obtain a concentration of 1 × 10^4 ^cells/ml, and 1 ml aliquots of the cell suspension were dispensed into 22-mm wells of 12-well plates (Falcon, Becton Dickinson and Co., Franklin Lakes, NJ). After a 10-min incubation at 37°C to allow collagen gelation, 1 ml defined medium was added above the gels. Collagen gel cultures were then incubated in defined medium for 6 days to allow the formation of cystic structures [12]. Thereafter, fresh defined medium was added with or without the indicated treatments and the gels were gently loosened from the wells by passing a curved-tip metallic spatula around their circumference and were allowed to float in the medium [90]. After 48 hours, medium and treatments were renewed and the cultures were incubated for an additional 48 hours.

For incorporation into fibrin gels, cells were suspended in a polymerizing fibrinogen solution prepared essentially as described [91]. Briefly, bovine fibrinogen (Cat. F-4753, Sigma) was dissolved at 37°C in calcium-free DMEM to obtain a final protein concentration of 2.5 mg/ml. J3B1A cells were suspended in the fibrinogen solution at a concentration of 1 × 10^4^ cells/ml, and clotting was initiated by adding 1/10 v/v of CaCl_2 _(2 mg/ml) and 25 U/ml of thrombin (Sigma, Cat. T4684). The mixture was immediately transferred into 16-mm wells (400 μl) and allowed to gel for at least 2 min at room temperature before being overlaid with defined medium. Aprotinin (Trasylol, Bayer Pharma, Zurich, Switzerland) was added to the medium at a concentration of 200 kallikrein inhibitory units/ml to prevent lysis of the fibrin substrate [91]. J3B1A cells were grown for 4–6 days to allow formation of small cysts and were subsequently incubated for an additional 7 days in the presence or absence of different concentrations of TGF-β1. For incorporation in laminin-rich gels, J3B1A cells were suspended in growth factor-reduced Matrigel (BD Bioscences) or in a 1:1 mixture of growth factor-reduced Matrigel and type I collagen. 20 μl drops were placed into 16-mm wells and allowed to solidify for 90 min at 37°C before being overlaid with 400 μl defined medium [92]. After a 6-day incubation to allow cyst formation, the cultures were treated with TGF-β1.

### Quantification of branching morphogenesis

At the indicated time points, 10 randomly selected colonies per experimental condition in each of at least three separate experiments (i.e., at least 30 colonies per experimental condition) were photographed under bright field illumination using the 10 × objective of a Nikon Diaphot TMD inverted photomicroscope. Quantification of branching morphogenesis was carried out by counting the number of radial outgrowths longer than 40 μm per colony. Data were expressed as mean number of outgrowths per colony ± s.e.m., and statistical significance was determined using the Student's unpaired *t*-test.

### Heat and acid treatment of FCS

Heating of FCS (GIBCO Invitrogen Ltd) was performed by diluting the serum to 10% in DMEM/F12 and heating at 70°C for 10 min. Acidification was carried out by bringing FCS to pH 3.0 with 0.5 M HCl. After 3 h incubation at room temperature, the pH was adjusted to 7.4 by the addition of 0.1 M sodium hydroxide.

### Plastic embedment and semi-thin sectioning

Collagen gel cultures prepared as described above and incubated for 4 days in the presence or absence of 50 pg/ml TGF-β1 were fixed *in situ *overnight with 2.5% glutaraldehyde in 100 mM sodium cacodylate buffer (pH 7.4). After extensive rinsing in the same buffer, the gels were gently removed from the wells and cut into 2 mm × 2 mm fragments. These were extensively rinsed in cacodylate buffer, post-fixed in 1% osmium tetroxide in Veronal acetate buffer for 45 min, stained *en bloc *with 2.5% uranyl acetate in 50% ethanol, dehydrated in graded ethanols and embedded in Epon 812 as described [4]. Semi-thin (1 μm-thick) sections were cut with an LKB Ultramicrotome (LKB Instruments, Gaithersburg, MD), stained with 1% methylene blue and photographed under transmitted light using an Axiophot photomicroscope (Carl Zeiss, Oberkaden, Germany).

### Northern blot hybridization

Confluent monolayers of J3B1A cells in defined medium were incubated with or without different concentrations of TGF-β1. After 24 or 48 hours, the dishes were washed with ice-cold PBS and total cellular RNA was extracted with Trizol reagent (Life Technologies, Paisley, Scotland) according to manufacturer's instructions. RNA was denatured with glyoxal, electrophoresed in a 1% agarose gel (15 μg RNA per lane), and transferred overnight onto nylon membranes (Hybond-N, Amersham, Buckinghamshire, UK). RNAs were crosslinked by baking the filters at 80°C for 2 h and stained with methylene blue to assess 18S and 28S ribosomal RNA integrity. Filters were hybridized for 16 h at 65°C with 1.5 × 10^6^ cpm/ml of ^32^P-labeled cRNA probes generated from mouse MMP-9, mouse MMP-2, or human MT1-MMP cDNAs (kindly provided by Dr. M. Pepper, Geneva, Switzerland). As an internal control for determining the amount of RNA loaded, the filters were simultaneously hybridized with a ^32^P-labeled P0 ribosomal phosphoprotein cRNA probe. Post-hybridization washes were performed as previously described [93]. Filters were exposed to Kodak XAR-5 films at -70°C between intensifying screens.

### Western blot analysis

J3B1A cells were plated in 100-mm dishes in defined medium at 2 × 10^6 ^cells/dish and grown to confluence. They were then left untreated or treated with 50 pg/ml, 200 pg/ml or 1 ng/ml TGF-β1. After 3 days, conditioned media (for MMP-9 and MMP-13 analyses) or total protein extracts (for MT1-MMP analyses) were collected. The conditioned media were supplemented with 0.5 mM phenylmethylsulfonyl fluoride (PMSF) and 15 mM HEPES, and centrifuged at 340 *g *for 5 minutes. The resulting supernatants were concentrated 3-fold by centrifugal filtration using a Centricon YM-10 cartridge (Amicon-Millipore, Volketsvil, Switzerland). For protein extracts, cells were washed in PBS and incubated in lysis buffer (50 mM Tris-HCl, 150 mM NaCl, 5 mM EDTA, pH 8.0, 1% Triton X-100 and 1% NP-40) containing 1 mM PMSF, 200 KIU Trasylol, 2 μg/ml leupeptin and 1 μg/ml pepstatin A for 1 h on ice. The lysate was centrifuged at 12'000 g for 15 min at 4°C, and the supernatant was collected. Equal amounts of concentrated conditioned media (15 μl) or protein extracts (10 μg) were separated by 10% SDS-PAGE before being transferred onto polyvinylidene difluoride membranes (PVDF, Bio-Rad, Reinach, Switzerland). Uniform loading of lanes was verified by silver staining of proteins. Nonspecific binding sites were blocked by incubating the membranes 90 min at room temperature in PBS containing 0.4% (vol/vol) Tween 20 (PBS-Tween) and 5% (wt/vol) non-fat milk powder. The membranes were then incubated overnight at 4°C with rabbit polyclonal antibody to MMP-9 (ab38898, Abcam Inc., Cambridge, MA; 1:5000 dilution), mouse monoclonal antibody to MMP-13 (Clone LIPCO IID1, Lab Vision Corporation, Fremont, CA; 1:200 dilution), or mouse monoclonal antibody to MT1-MMP (Ab-4, Oncogene, San Diego, CA; 1:2000 dilution). After extensive washing in PBS-Tween, the membranes were incubated for 1 hour at room temperature with horseradish peroxydase-conjugated secondary antibodies (Amersham Biosciences, Otelfingen, Switzerland), diluted 1:3000. Membranes were then washed extensively in blocking buffer and antigen-antibody complexes detected by enhanced chemiluminescence, according to the manufacturer's instructions (Amersham). Conditioned media from PMA-treated U937 cells, which produce MMP-9 [94], and from SVEC4-10 cells, which express MMP-13 and MMP-14 [95], were used as positive controls.

### Gelatin zymography

J3B1A cells were plated in 60-mm dishes in defined medium at 2 × 10^6 ^cells/dish and grown for 24 hours. They were then left untreated or treated with 50 pg/ml, 200 pg/ml or 1 ng/ml TGF-β1. After 3 days, conditioned media were collected, supplemented with 0.5 mM PMSF and 15 mM HEPES, and centrifuged at 340 *g *for 5 minutes. The resulting supernatants were stored at -20°C until use, then mixed with an equal volume of 2 × Novex SDS sample buffer (Invitrogen, Bern, Switzerland) without reducing agents and the mixture was loaded (10 μl per lane) on a precast 0.1% gelatin-10% acrylamide zymography gel (Invitrogen). After electrophoresis at 4°C, the gels were soaked in 2.5% Triton X-100 for 20 minutes to remove SDS, incubated overnight at 37°C in reaction buffer (50 mM Tris-HCl pH 7.4, containing 150 mM NaCl, 10 mM CaCl2), and then stained with methanol: acetic acid: water (30:10:60) containing 0.25% Coomassie Blue R250 for 4 hours. Gelatinolytic activity was detected as a clear band on a background of uniform blue staining. Molecular masses of gelatinolytic bands were estimated with pre-stained molecular mass markers (Bio-Rad Laboratories AG, Reinach, Switzerland). Conditioned medium from PMA-treated human U937 cells, which are known to produce MMP-2 and MMP-9 [94], was used as a positive control.

## Authors' contributions

RM was responsible for the conception and design of the study, for the supervision of experiments performed by a technician, for the analysis, quantitation and interpretation of data, for figure preparation, and for manuscript drafting. FC contributed to the acquisition of data (immunofluorescence analysis of epithelial and mesenchymal markers) and their interpretation. PS contributed to the acquisition of data (Northern blot, Western blot and zymographic analysis of MMPs) and to their interpretation. FC and PS critically read the manuscript and all authors approved the final version.
